# The association between smoking and the occurrence of hyperuricemia: A retrospective cohort study

**DOI:** 10.18332/tid/204253

**Published:** 2025-05-30

**Authors:** Peihua Li, Xinyu Li, Guosheng Li, Bing Wang, Yudan Liu, Yuedong Zhao, Qing Yu, Zhengnan Gao, Xuhan Liu

**Affiliations:** 1 Department of Endocrinology, Dalian Municipal Central Hospital, Dalian, China; 2 Department of Pathogenic Microbiology, College of Basic Medical Sciences, Jinzhou Medical University, Jinzhou, China; 3 Laboratory Pathology Department, Ningbo Clinical Pathology Diagnosis Center, Ningbo, China; 4 Department of Neuroendocrine Pharmacology, School of Pharmacy, China Medical University, Shenyang, China

**Keywords:** smoking, smoking cessation, hyperuricemia, uric acid, cohort study

## Abstract

**INTRODUCTION:**

A retrospective cohort study was conducted to study the association between smoking and hyperuricemia (HUA).

**METHODS:**

By collecting and analyzing clinical data of 3196 patients with undiagnosed HUA at baseline in Dalian Municipal Central Hospital of China between 1 January 2010 and 1 January 2021, patients were grouped according to baseline smoking status and smoking index (the number of cigarettes smoked per day × number of years of smoking). Cox regression analysis was used to perform univariable and multivariable analyses of factors that may influence the occurrence of HUA. And further stratification was performed.

**RESULTS:**

The median follow-up time was 3.62 years. A total of 485 (15.2%) patients developed HUA (≥420 μmol/L). The incidence of HUA was significantly higher in the smoking group than in the non-smoking group (p<0.05). There was a statistically significant difference in the incidence of HUA between the smoking index 1–4 (>0) groups and the smoking index 0 (0) group (p<0.05). Multifactorial Cox regression analyses were performed separately and after adjustment for relevant influences, the results showed that smoking was an independent risk factor for the occurrence of HUA with a hazard ratio (HR) of 1.38 (95% CI: 1.11–1.72). And the smoking index groups 401–600 and ≥601 were independent risk factors for the occurrence of HUA, with HRs of 1.46 (95% CI: 1.20–1.70) and 1.53 (95% CI: 1.06–2.22), respectively. The further stratified analysis revealed that smoking remained an independent risk factor for the occurrence of HUA in all subgroups, and the smoking index ≥601 group was also an independent risk factor for the occurrence of HUA, with HRs greater than 1 (p<0.05).

**CONCLUSIONS:**

Smoking is an independent risk factor for the occurrence of HUA and is independent of gender, whether a woman is menopausal, body mass index (BMI), and alcohol consumption. The smoking index ≥601 was an independent risk factor for the occurrence of HUA.

## INTRODUCTION

Hyperuricemia (HUA) is a metabolic disease caused by abnormal purine metabolism resulting in abnormally high blood uric acid levels, and several studies have confirmed that baseline uric acid levels are a major predictor of gout attacks^1^. Much evidence suggests that hyperuricemia and gout are independent risk factors for diseases such as diabetes mellitus and cardiovascular disease, and are independent predictors of premature death in patients^2^. In recent years, with the improvement in living standards, the incidence of hyperuricemia in China has been increasing year by year, and a meta-analysis showed that the overall prevalence of HUA in China is 13.3%^3^. The progression from asymptomatic hyperuricemia to gout is a continuous and progressive pathophysiological and clinical process, so early detection, early treatment, and long-term monitoring of blood uric acid levels are important to improve the prognosis of patients.

Hyperuricemia is a disease caused by the interaction of both genetic and environmental factors. The enzyme allantoin is present in mammals and breaks down uric acid into allantoin, whereas during natural evolution, humans have lost the functional gene encoding allantoin through natural selection by genetic mutation and therefore cannot break down uric acid into soluble allantoin and thus excrete it from the body^4^. Thus, blood uric acid levels in humans are much higher than in other animals carrying uricase. Currently, the risk factors for hyperuricemia that have been identified are gender (male), increasing age, elevated body mass index (BMI), excessive purine dietary intake, alcohol consumption, fructose intake, and so on^5^.

Smoking is an important social issue and is often considered a risk factor for many well-known chronic diseases such as cancer^6^, and it is also thought to be associated with several chronic musculoskeletal disorders such as rheumatoid arthritis^7^. Given the systemic, pro-inflammatory, and potentially anti-inflammatory effects of smoking, and the role of smoking as a risk factor for other gout-related disorders, it is reasonable to assume that smoking has an impact on the risk of developing HUA. In this retrospective cohort study, we analyzed the association between smoking and the occurrence of HUA by retrospectively analyzing cases of patients who were not diagnosed with HUA at baseline during at least 2 hospitalizations in Dalian Municipal Central Hospital between 1 January 2010 and 1 January 2021, and grouped them according to different smoking status and smoking index at baseline, to clarify whether smoking was an independent influencing factor for the occurrence of HUA.

## METHODS

### Study design

This retrospective cohort study was conducted at Dalian Municipal Central Hospital in China, between 1 January 2010 and 1 January 2021. The median follow-up time for all patients was 3.62 years. A Cox proportional risk model was constructed: start time was defined as the time of first admission, end time as the time of last admission, and end event as the occurrence of HUA. Univariable analyses of factors that might influence the occurrence of HUA were first performed to screen out statistically significant factors (p<0.1) and to assess the proportional risk assumption. A multifactorial analysis was then performed on the screened factors to determine whether smoking was an independent factor influencing the occurrence of HUA (p<0.05). Finally, further stratified analysis was performed based on the statistically significant factors other than smoking in the multifactorial analysis, and subgroups were divided according to baseline clinical data, and Cox models were constructed to adjust for other relevant factors (p<0.1) to clarify whether smoking was an independent factor for the occurrence of HUA.

### Study population

Our data are sourced from the Dalian Municipal Central Hospital database, which contains clinical data on patients who have visited our outpatient clinic or have been hospitalized for various reasons. Given the large amount of missing clinical data from outpatients, we chose to collect and analyze data from inpatients to definitively investigate the association between smoking and HUA.

**Figure 1:**
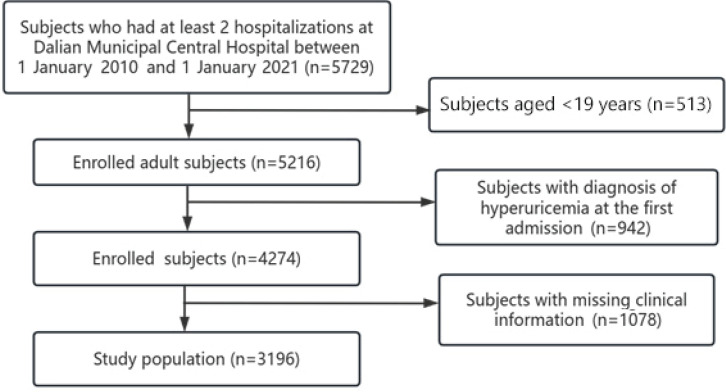
Flow chart showing study population, Dalian Municipal Central Hospital, China, 1 January 2010 – 1 January 2021

Patients with at least 2 hospitalizations in Dalian Municipal Central Hospital between 1 January 2010 and 1 January 2021, with an interval of more than 1 year between the first and the last admission, with a blood uric acid level of <420 μmol/L at the time of the first admission, without a diagnosis of HUA, and with a blood uric acid test at the time of the last hospitalization, were selected. Inclusion criteria: aged ≥18 years, those with a time interval of more than 1 year between the first admission and the last admission, blood uric acid level <420 μmol/L at the first admission, and no diagnosis of hyperuricemia, those with complete information on clinical data. Exclusion criteria: patients with pregnancy, malignancy, rheumatoid arthritis or gout, those with combined acute or chronic infections, those with hematologic disorders, cardiopulmonary failure, abnormal liver, and kidney function, and severe diseases of other systems, those taking drugs affecting uric acid metabolism within the previous 12 months and those with missing clinical information. According to the above inclusion and exclusion criteria, a total of 3196 patients were included in this study (Figure 1).

### Data source

Applying the Yidu Cloud electronic medical record retrieval system, we collected patients’ personal history such as past disease history, medication history, smoking history, alcohol history, height, weight, blood pressure, BMI, and laboratory indexes such as TC, TG, LDL-C, HDL-C, SUA, BUN, SCr, ALT, AST, and γ-GT according to the above-mentioned nadir criteria. All patients fasted overnight for 12 hours or more, and morning arm venous blood was collected from patients. The four lipid items (HDL-C, TC, LDL-C, and TG), ALT, AST, γ-GT, BUN, SCr, and SUA were measured using an automated biochemical analyzer (Siemens, Germany, ADVIA2400 biochemical system). Blood pressure and pulse rate measurement method: the blood pressure and pulse rate were measured in the right upper arm in the seated position while the patient was at rest, with a 5-minute interval between each measurement, and a total of 3 measurements were taken and averaged.

### Baseline measurements and definitions

The groups were grouped according to baseline smoking status and divided into three groups: smoking, non-smoking, and smoking cessation. Smoking: this refers to smoking at least one cigarette per day (continuously or intermittently) for more than 6 months during their lifetime and still smoking during the 30 days before the survey. Smoking cessation (former smokers): smoked at least one cigarette per day (continuously or intermittently) for more than 6 months during their lifetime, but had stopped smoking 30 days before the baseline survey. Non-smoking: continuous or intermittent smoking for less than 6 months or not being in a passive smoking environment within 30 days of the baseline survey^8^.

The smoking index (the number of cigarettes smoked per day × number of years of smoking) was divided into five groups according to the international standard: group 0: 0 points, group 1: 1–200 points, group 2: 201–400 points, group 3: 401–600 points, and group 4: ≥601 points. Alcohol consumption: <1 year of drinking and abstinence; non-drinking: never drinking and ≥1 year of abstinence.

The diagnosis of hyperuricemia was based on the Chinese guidelines for the diagnosis and treatment of hyperuricemia and gout (2019). Diagnosis of diabetes mellitus was based on the Chinese guidelines for the prevention and treatment of type 2 diabetes mellitus (2020). Hypertension was diagnosed according to the 7th report of the US Joint National Committee on Prevention, Detection, Evaluation, and Treatment of Hypertension (JNC7)^9^.

### Outcome measurement and covariates

Age, BMI, systolic blood pressure, diastolic blood pressure, heart rate, ALT, AST, γ-GT, TC, TG, HDL-C, LDL-C, SCr, BUN, and SUA were observed at baseline data, and the changes in blood uric acid level after follow-up. We mainly analyzed the correlation between different smoking statuses and smoking index and the occurrence of HUA.

### Statistical analysis

Data were statistically analyzed using SPSS 23.0 software, and all data were tested for normality, in the normality test, p>0.05 was considered that the data obeyed normal distribution, with measurement data conforming to a normal distribution expressed as mean and standard deviation (SD), and for a non-normal distribution were expressed as median and interquartile range (IQR). Count data were expressed frequencies (n) and percentages (%). One-way ANOVA was used for group comparisons of normally distributed data, and the Kruskal-Wallis H test was used for non-normally distributed data. The χ^2^ test was used for the comparison of rates. The follow-up data were analyzed by regression using the Cox proportional risk model method. Cox models are reported as hazard ratios (HRs) and 95% CI. All tests were two-tailed. A p<0.05 was statistically significant.

## RESULTS

### Comparison of general clinical characteristics at baseline

Comparing the general clinical characteristics of the smoking status groups, it was found that there were significant differences in the distribution of age, ALT, γ-GT, TC, HDL-C, and LDL-C among the study subjects in different smoking statuses, and the differences were statistically significant (p<0.05). While there were no significant differences in the distribution of BMI, systolic blood pressure, diastolic blood pressure, heart rate, AST, TG, SCr, BUN, SUA, history of hypertension, history of diabetes mellitus, and history of alcohol consumption, and none of the differences were statistically significant (p>0.05) (Table 1).

**Table 1:** Comparison of the general clinical characteristics of the groups for different smoking status, Dalian Municipal Central Hospital, China, 1 January 2010 – 1 January 2021 (N=3196)

*Characteristics*	*Non-smoking* *(N=1400)*	*Smoking* *(N=1200)*	*Smoking cessation* *(N=596)*	
	*Mean ± SD*	*Mean ± SD*	*Mean ± SD*	*p ^a^*
Age (years)	60.83 ± 12.35	63.76 ± 6.99	62.89 ± 8.76	0.015*
BMI (kg/m^2^)	25.85 ± 3.61	25.59 ± 3.50	25.85 ± 3.44	0.129
SBP (mmHg)	143.04 ± 21.79	142.03 ± 20.00	141.50 ± 21.72	0.332
DBP (mmHg)	79.76 ± 11.28	79.07 ± 11.80	78.91 ± 12.12	0.911
HR (bpm)	78.09 ± 10.88	78.16 ± 10.88	77.66 ± 11.05	0.787
	***Median (IQR)***	***Median (IQR)***	***Median (IQR)***	***p ^b^***
ALT (U/L)	18 (13.25–24.00)	19 (13.00–28.00)	21 (15.00–28.25)	<0.001*
AST (U/L)	20 (16.00–24.75)	20 (16.00–25.00)	20 (16.00–25.00)	0.673
γ-GT (U/L)	23 (16.00–33.00)	29 (20.00–38.00)	29 (20.00–36.00)	<0.001*
TC (mmol/L)	5.24 (4.58–5.94)	5.17 (4.45–5.84)	5.015 (4.33–5.75)	<0.001*
TG (mmol/L)	1.435 (1.08–1.98)	1.39 (1.10–2.07)	1.345 (1.12–2.00)	0.624
HDL-C (mmol/L)	1.22 (1.00–1.45)	1.145 (0.95–1.39)	1.11 (0.92–1.40)	<0.001*
LDL-C (mmol/L)	3.14 (2.57–3.75)	3.05 (2.70–3.70)	3.055 (2.45–3.67)	0.048*
SCr (μmol/L)	58.1 (51.80–64.00)	59.0 (54.2–63.4)	58.8 (54.0–63.4)	0.236
BUN (mmol/L)	5.27 (4.47–6.41)	5.42 (4.34–7.07)	5.5 (4.35–7.18)	0.646
SUA (μmol/L)	325 (285–366)	335 (288–371)	330 (288–372)	0.161
	***n (%)***	***n (%)***	***n (%)***	***p ^c^***
Hypertension	280 (20.0)	275 (22.9)	130 (21.8)	0.189
Diabetes mellitus	324 (23.1)	305 (25.4)	144 (24.2)	0.402
Drinking	409 (29.2)	396 (33.0)	188 (31.5)	0.111

a One-way ANOVA was used for group comparisons.

b The Kruskal-Wallis H test was used for group comparisons.

c The χ^2^ test was used for the comparison of rates.

* p<0.05 is significant. IQR: interquartile range.

The study subjects were divided into five groups based on the smoking index. When the SUA of the smoking index groups were compared, it was found that there was a significant difference in the distribution of SUA among the subjects in the five groups, and the difference was statistically significant (p<0.001). Group 0 (0), group 1 (1–200), group 2 (201–400), group 3 (401–600) and group 4 (≥601) had SUA of 304 (252–362), 308.5 (258.5–362), 310 (264–354), 322 (281–362) and 327 (276–367), respectively.

### Incidence rates of HUA

The median follow-up time for all patients was 3.62 years, and a total of 485 patients developed HUA, with an overall HUA incidence of 15.2%. The incidence of HUA was 16.5% in men and 13.2% in women (p=0.012).

Comparison of the incidence of HUA among groups with different smoking statuses was: smoking group 21.0%, smoking cessation group 12.0% vs non-smoking group10.9% (p<0.001, p=0.486, respectively). Comparison of the incidence of HUA in men was: smoking group 22.2%, smoking cessation group 12.8% vs non-smoking group 10.9% (p<0.001, p=0.333, respectively). Comparison of the incidence of HUA in women was: smoking group 17.9%, smoking cessation group 11.4% vs non-smoking group 11.1% (p=0.003, p=0.436, respectively) (Supplementary file Table S1).

For the comparison of the incidence of HUA among groups with different smoking index, the study subjects were divided into five groups based on the smoking index: 0 (0), 1 (1–200), 2 (201–400), 3 (401–600), and 4 (≥601). The study subjects in each group were 1996 (62.5%), 193 (6.0%), 272 (8.5%), 209 (6.5%), and 526 (16.5%), respectively. The incidence of HUA was 11.7%, 16.6%, 18.4%, 21.5%, and 23.8%, respectively. The incidence of HUA was statistically different for each of groups 1 (1–200), 2 (201–400), 3 (401–600), and 4 (≥601) vs group 0 (0) (all p<0.05) (Supplementary file Table S2).

### Cox regression analysis

Univariable analysis of factors that may influence the occurrence of HUA was first performed to screen for statistically significant influences (p<0.1). Cox univariable analysis showed that smoking, smoking index group 1–4 (>0), age, gender, BMI, systolic blood pressure, TG, HDL-C, history of hypertension, and alcohol consumption were influential factors for the occurrence of HUA (p<0.1). All variables satisfy the proportional risk assumption test (Table 2).

**Table 2:** Cox regression univariable and multivariable analysis of smoking status and HUA occurrence, Dalian Municipal Central Hospital, China, 1 January 2010 – 1 January 2021 (N=3196)

*Variables*	*Univariable*		*Multivariable*	
	*HR (95% CI)*	*p*	*AHR (95% CI)*	*p*
Age (years)	1.34 (1.00–1.80)	0.049*	1.31 (0.97–1.76)	0.081
Gender (male)	1.47 (1.16–1.87)	0.002*	1.37 (1.13–1.65)	0.001**
BMI (kg/m^2^)	1.33 (1.09–1.62)	0.005*	1.32 (1.08–1.61)	0.006**
SBP (mmHg)	1.01 (1.00–1.02)	0.036*	1.01 (1.00–1.01)	0.169
DBP (mmHg)	1.00 (0.92–1.09)	0.952		
HR (bpm)	1.00 (0.99–1.01)	0.832		
ALT (U/L)	1.00 (1.00–1.01)	0.297		
AST (U/L)	1.00 (1.00–1.01)	0.613		
γ-GT (U/L)	1.00 (0.92–1.09)	0.93		
TC (mmol/L)	1.02 (0.77–1.35)	0.903		
TG (mmol/L)	1.05 (1.01–1.09)	0.026*	1.04 (1.00–1.09)	0.057
HDL-C (mmol/L)	0.63 (0.47–0.85)	0.003*	0.68 (0.46–1.01)	0.055
LDL-C (mmol/L)	1.02 (0.71–1.46)	0.926		
SCr (μmol/L)	1.00 (1.00–1.01)	0.236		
BUN (mmol/L)	1.01 (1.00–1.01)	0.31		
Hypertension	1.41 (1.06–1.88)	0.02*	1.35 (0.86–2.11)	0.195
Diabetes mellitus	1.20 (0.92–1.58)	0.188		
Drinking	1.43 (1.15–1.77)	0.001*	1.40 (1.14–1.70)	0.001**
**Smoking status**				
Non-smoking ®	1		1	
Smoking	1.72 (1.41–2.10)	<0.001*	1.38 (1.11–1.72)	0.004**
Smoking cessation	1.33 (0.99–1.78)	0.059*	1.15 (0.86–1.54)	0.333

AHR: adjusted hazard ratio. Multivariable analysis adjusted for age, gender, BMI, SBP, TG, HDL-C, history of hypertension, and drinking at p<0.1 in the Cox regression univariable analysis.

* Results of univariable analysis p<0.1 is significant.

** Results of multivariable analysis p<0.05 is significant. ® Reference category.

After correcting for age, gender, BMI, systolic blood pressure, TG, HDL-C, history of hypertension, and alcohol consumption at p<0.1 in the Cox regression univariable analysis, the Cox multifactor analysis showed that smoking was an independent risk factor for the occurrence of HUA when grouped according to smoking status, with an adjusted hazard ratio (AHR) of 1.38 (95% CI: 1.11–1.72), with a statistically significant difference (p<0.05). And gender (AHR=1.37; 95% CI: 1.13–1.65), BMI (AHR=1.32; 95% CI: 1.08–1.61), and alcohol consumption (AHR=1.39; 95% CI: 1.14–1.70) were also found to be independent risk factors for the occurrence of HUA, and the differences were statistically significant (p<0.05) (Table 2).

The Cox multifactor analysis also showed that smoking index groups 3 (401–600) and 4 (≥601) were independent risk factors for the occurrence of HUA according to smoking index grouping, with AHRs of 1.46 (95% CI: 1.20–1.70) and 1.53 (95% CI: 1.06–2.22), respectively, with statistically significant differences (p<0.05) (Table 3).

**Table 3:** Cox regression univariable and multivariable analysis of smoking index and HUA occurrence, Dalian Municipal Central Hospital, China, 1 January 2010 – 1 January 2021 (N=3196)

*Variables*	*Univariable*		*Multivariable*	
	*HR (95% CI)*	*p*	*AHR (95% CI)*	*p*
**Smoking index**				
0 (0) ®	1		1	
1 (1–200)	1.51 (1.06–2.54)	0.02*	1.38 (0.94–2.02)	0.102
2 (201–400)	1.78 (1.25–2.52)	0.001*	1.39 (1.00–1.93)	0.051
3 (401–600)	1.93 (1.44–2.59)	<0.001*	1.46 (1.20–1.70)	0.03**
4 (≥601)	1.95 (1.41–2.70)	<0.001*	1.53 (1.06–2.22)	0.024**

AHR: adjusted hazard ratio. Multivariable analysis adjusted for age, gender, BMI, SBP, TG, HDL-C, history of hypertension, and drinking at p<0.1 in the Cox regression univariable analysis.

* Results of univariable analysis p<0.1 is significant.

** Results of multivariable analysis p<0.05 is significant. ® Reference category.

### Stratification analysis

In the Cox regression multivariable analysis, gender, BMI, and alcohol consumption were also found to be independent risk factors for HUA, and the differences were statistically significant (p<0.05). Therefore, further stratified analyses were performed on gender, whether women were menopausal or not, BMI and alcohol consumption, and baseline clinical data were divided into various subgroups, and Cox models were constructed separately, adjusting for other relevant influencing factors to clarify whether smoking was an independent influencing factor on the occurrence of HUA.

### Gender stratification

Gender was stratified into two groups, male and female. The results of the Cox multifactor analysis showed that group according to smoking status, after adjusting for other relevant influencing factors, both in the male population (AHR=1.55; 95% CI: 1.16–2.08, p<0.05) and the female population (AHR=1.48; 95% CI: 1.02–2.15, p<0.05), smoking was a risk factor for the occurrence of HUA (Table 4).

**Table 4:** Multivariable Cox regression analysis of smoking status and HUA occurrence stratified by gender, women for menopause or not, BMI and alcohol consumption history, Dalian Municipal Central Hospital, China, 1 January 2010 – 1 January 2021 (N=3196)

*Variables*	*Smoking status*	*AHR (95% CI)*	*p*
Male	Non-smoking ®	1	
	Smoking	1.55 (1.16–2.08)^a^	0.003*
	Smoking cessation	1.37 (0.90–2.08)^a^	0.138
Female	Non-smoking ®	1	
	Smoking	1.48 (1.02–2.15)^a^	0.037*
	Smoking cessation	1.30 (0.69–2.45)^a^	0.421
Nonmenopausal	Non-smoking ®	1	
	Smoking	1.48 (1.03–2.12)^a^	0.035*
	Smoking cessation	1.35 (0.67–2.74)^a^	0.404
Menopausal	Non-smoking ®	1	
	Smoking	1.42 (1.12–1.81)^a^	0.004*
	Smoking cessation	1.39 (0.58–3.35)^a^	0.457
BMI <25 kg/m^2^	Non-smoking ®	1	
	Smoking	1.40 (1.07–1.82)^b^	0.013*
	Smoking cessation	1.41 (0.93–2.13)^b^	0.104
BMI ≥25 kg/m^2^	Non-smoking ®	1	
	Smoking	1.69 (1.13–2.55)^b^	0.011*
	Smoking cessation	1.62 (0.87–3.02)^b^	0.132
Drinking	Non-smoking ®	1	
	Smoking	1.82 (1.32–2.50)^c^	<0.001*
	Smoking cessation	1.35 (0.65–2.81)^c^	0.417
Non-drinking	Non-smoking ®	1	
	Smoking	1.69 (1.05–2.72)^c^	0.03*
	Smoking cessation	1.28 (0.94–1.75)^c^	0.119

AHR: adjusted hazard ratio.

a Multivariable analysis adjusted for age, BMI, SBP, TG, HDL-C, history of hypertension, and drinking at p<0.1 in the Cox regression univariable analysis.

b Multivariable analysis adjusted for age, gender, SBP, TG, HDL-C, history of hypertension, and drinking at p<0.1 in the Cox regression univariable analysis.

c Multivariable analysis adjusted for age, gender, BMI, SBP, TG, HDL-C, and history of hypertension at p<0.1 in the Cox regression univariable analysis.

* p<0.05 is significant. ® Reference category.

Grouped according to the smoking index, after adjusting for other relevant influencing factors, in the male population, smoking index group 3 (401–600) (AHR=1.65; 95% CI: 1.13–2.43, p<0.05) and group 4 (≥601) (AHR=1.89; 95% CI: 1.26–2.86, p<0.05) were risk factors influencing the occurrence of HUA. In the female population, smoking index group 4 (≥601) was a risk factor for the occurrence of HUA (AHR=1.76; 95% CI: 1.16–2.65, p<0.05) (Table 5).

**Table 5:** Multivariable Cox regression analysis of smoking index and HUA occurrence stratified by gender, women for menopause or not, BMI and alcohol consumption history, Dalian Municipal Central Hospital, China, 1 January 2010 – 1 January 2021 (N=3196)

*Variables*	*Smoking index*	*AHR (95% CI)*	*p*
Male	0 (0) ®	1	
	1 (1–200)	1.15 (0.83–1.60)^a^	0.407
	2 (201–400)	1.49 (0.94–2.37)^a^	0.092
	3 (401–600)	1.65 (1.13–2.43)^a^	0.01*
	4 (≥601)	1.89 (1.26–2.86)^a^	0.002*
Female	0 (0) ®	1	
	1 (1–200)	1.02 (0.51–2.03)^a^	0.961
	2 (201–400)	1.25 (0.88–1.76)^a^	0.209
	3 (401–600)	1.48 (0.92–2.36)^a^	0.104
	4 (≥601)	1.76 (1.16–2.65)^a^	0.007*
Non-menopausal	0 (0) ®	1	
	1 (1–200)	1.05 (0.24–4.56)^a^	0.948
	2 (201–400)	1.11 (0.26–4.72)^a^	0.888
	3 (401–600)	1.44 (0.50–4.19)^a^	0.499
	4 (≥601)	1.50 (1.04–2.17)^a^	0.029*
Menopausal	0 (0) ®	1	
	1 (1–200)	1.12 (0.81–1.55)^a^	0.499
	2 (201–400)	1.34 (0.86–2.09)^a^	0.201
	3 (401–600)	1.36 (0.86–2.15)^a^	0.185
	4 (≥601)	1.60 (1.21–2.13)^a^	0.001*
BMI <25 kg/m^2^	0 (0) ®	1	
	1 (1–200)	1.11 (0.52–2.35)^b^	0.785
	2 (201–400)	1.29 (0.81–2.05)^b^	0.286
	3 (401–600)	1.54 (0.99–2.40)^b^	0.058
	4 (≥601)	1.74 (1.21–2.50)^b^	0.003*
BMI ≥25 kg/m^2^	0 (0) ®	1	
	1 (1–200)	1.28 (0.93–1.75) ^b^	0.129
	2 (201–400)	1.54 (0.96–2.46)^b^	0.072
	3 (401–600)	1.60 (0.86–2.98)^b^	0.139
	4 (≥601)	1.80 (1.01–3.21)^b^	0.047*
Drinking	0 (0) ®	1	
	1 (1–200)	1.24 (0.76–2.03)^c^	0.393
	2 (201–400)	1.25 (0.77–2.05)^c^	0.364
	3 (401–600)	1.65 (0.99–2.73)^c^	0.054
	4 (≥601)	1.85 (1.11–3.07)^c^	0.018*
Non-drinking	0 (0) ®	1	
	1 (1–200)	1.16 (0.67–2.00)^c^	0.603
	2 (201–400)	1.22 (0.79–1.89)^c^	0.375
	3 (401–600)	1.31 (0.90–1.90)^c^	0.157
	4 (≥601)	1.71 (1.16–2.52)^c^	0.006*

AHR: adjusted hazard ratio.

a Multivariable analysis adjusted for age, BMI, SBP, TG, HDL-C, history of hypertension, and drinking at p<0.1 in the Cox regression univariable analysis.

b Multivariable analysis adjusted for age, gender, SBP, TG, HDL-C, history of hypertension, and drinking at p<0.1 in the Cox regression univariable analysis.

c Multivariable analysis adjusted for age, gender, BMI, SBP, TG, HDL-C, and history of hypertension at p<0.1 in the Cox regression univariable analysis.

* p<0.05 is significant. ® Reference category.

### Female menopause stratification

Women were stratified into two groups: non-menopausal and menopausal. The results of the Cox multifactor analysis showed that group according to smoking status, after adjusting for other relevant influencing factors, both in the population of non-menopausal women (AHR=1.48; 95% CI: 1.03–2.12, p<0.05) and the population of menopausal women (AHR=1.42; 95% CI: 1.12–1.81, p<0.05), smoking was a risk factor for the occurrence of HUA (Table 4).

Grouped according to the smoking index, after adjusting for other relevant influencing factors, both in the population of non-menopausal women (AHR=1.50; 95% CI: 1.04–2.17, p<0.05) and the population of menopausal women (AHR=1.60; 95% CI: 1.21–2.13, p<0.05), smoking index group 4 (≥601) was a risk factor for the occurrence of HUA (Table 5).

### BMI stratification

BMI was stratified into two groups, BMI <25 kg/m^2^ and BMI ≥25 kg/m^2^ (overweight). The results of the Cox multifactor analysis showed that group according to smoking status, after adjusting other relevant influencing factors, both in the population of BMI <25 kg/m^2^ (AHR=1.40; 95% CI: 1.07–1.82, p<0.05) and the population with BMI ≥25 kg/m^2^ (AHR=1.69; 95% CI: 1.13–2.55, p<0.05), smoking was a risk factor for the occurrence of HUA (Table 4).

Grouped according to the smoking index, after adjusting for other relevant influencing factors, both in the population of BMI <25 kg/m^2^ (AHR=1.74; 95% CI: 1.21–2.50, p<0.05) and the population with BMI ≥25 kg/m^2^ (AHR=1.80; 95% CI: 1.01–3.21, p<0.05), smoking index group 4 (≥601) was a risk factor affecting the occurrence of HUA (Table 5).

### Alcohol consumption stratification

Alcohol consumption was stratified into two groups, drinking and non-drinking. The results of the Cox multifactor analysis showed that grouped according to smoking status, after adjusting other relevant influencing factors, both in the population of drinking (AHR=1.82; 95% CI: 1.32–2.50, p<0.05) and the population of non-drinking (AHR=1.69; 95% CI: 1.05–2.72, p<0.05), smoking was a risk factor affecting the occurrence of HUA (Table 4).

Grouped according to the smoking index, after adjusting for other relevant influencing factors, both in the population of drinking (AHR=1.85; 95% CI: 1.11–3.07, p<0.05) and the population of non-drinking (AHR=1.71; 95% CI: 1.16–2.52, p<0.05), smoking index group 4 (≥601) was a risk factor influencing the occurrence of HUA (Table 5).

## DISCUSSION

We used the database of Dalian Municipal Central Hospital and included 3196 patients with undiagnosed HUA at baseline according to the inclusion and exclusion criteria. Relevant clinical data were retrospectively collected and analyzed, and the occurrence of HUA was followed to clearly study the association between smoking and HUA. HUA in both men and women is defined as a fasting blood uric acid level ≥420 μmol/L (or 7.0 mg/dL). By constructing a multifactorial Cox proportional risk model, after adjusting for other relevant influencing factors, we found that the incidence of HUA increased by 37.7% in smokers compared with non-smokers, and by 53.4% in smokers with smoking index ≥601 compared with non-smokers, and was not affected by sex, whether a woman was menopausal, BMI, and alcohol consumption. Smoking is an independent risk factor for the occurrence of HUA. And the risk of HUA was found to increase gradually with the increase in the smoking index. A smoking index ≥601 was an independent risk factor for the occurrence of HUA.

Smoking is one of the most important modifiable risk factors for many chronic diseases and death^10^, and smoking can damage almost all organs and systems of the body^11^. Current studies related to risk factors for the occurrence of hyperuricemia have focused on gender, age, obesity, exercise, diet, and alcohol consumption^12^. However, in a large number of studies on the potential impact of blood uric acid levels and the risk of hyperuricemia, smoking is usually considered a covariate, and the association between smoking and the occurrence of HUA is also still controversial in international clinical studies so far, and there is no consistent conclusion yet. Therefore, this article, using smoking as the main variable aims to investigate the association between smoking and hyperuricemia.

Most of the current studies on the association between smoking and hyperuricemia are cross-sectional studies, and the results are not yet consistent. Some studies have shown an association between smoking and hyperuricemia in the overall population^13^. Kim and Choe^14^ found a positive association between blood uric acid levels and smoking status only in female subjects, while there was no significant association among men. Another study showed that active and passive smoking were positively associated with blood uric acid levels only in women, and a dose-response relationship between smoking and the risk of hyperuricemia was found only in women, with no significant effect found in men^15^. Results from a study of patients with cardiovascular disease (CVD)^16^ showed that smokers had significantly higher blood uric acid levels than non-smokers. A study that examined the short-term effects of smoking on blood uric acid levels found that current smokers had a significant decrease in mean blood uric acid levels after 5 minutes of smoking a cigarette^17^. In two other cross-sectional studies of the Chinese population^18^ and of the middle-aged and elderly population^19^, after adjusting for potential confounders, smoking and HUA were found to be negatively associated with the overall population and the male population, while no significant association was observed in women.

There are relatively few follow-up studies on smoking and hyperuricemia and gout, and the results have not been consistently conclusive. The Framingham Heart Study (FHS) collected and analyzed longitudinal data from gout-free individuals at baseline and followed up at multiple time points (median follow-up time 37 years) and found that smoking reduced the risk of gout in the overall population and men^20^. An Atherosclerosis Risk in Communities (ARIC) study of older adults (aged ≥65 years) without gout at baseline found that smoking elevated the risk of developing gout in the overall population and women^21^. A non-significant correlation between smoking and an increased incidence of hyperuricemia was found in another Atherosclerosis Risk in Communities (ARIC) study^22^, and a non-significant correlation between smoking and an increased incidence of gout was also shown in a five-year prospective cohort study^23^.

However, the pathophysiological mechanisms by which smoking promotes the occurrence of HUA are not clear. Uric acid is the end product of purine catabolism in the body and is mainly produced from hypoxanthine and xanthine catalyzed by xanthine oxidase^24^. Studies have shown that elevated levels of xanthine oxidase due to elevated malondialdehyde levels in the serum of smokers may lead to increased synthesis of uric acid; on the other hand, elevated levels of xanthine oxidase may lead to increased production of reactive oxygen species, which in turn increases lipid peroxidation reactions, aggravating the great damage to cells increasing cell turnover thus leading to increased purine catabolism and thus increased uric acid production^25^. It has also been shown that the hypoxanthine-guanine phosphoribosyltransferase (HGPRT) gene in smokers has a higher frequency of reversal and a lower frequency of conversion compared to the corresponding gene in non-smokers, suggesting that cigarette smoke may induce adducts at guanine bases in the non-transcribed DNA strand, leading to an increased frequency of HGPRT gene mutations in smokers^26^ and that frequent HGPRT gene mutations in smokers may lead to decreased enzyme activity, resulting in increased hypoxanthine and guanine levels and thus increased blood uric acid levels^27^.

### Limitations

There are some limitations in this study. Firstly, this study was a retrospective study, and clinical information related to hospitalized patients was obtained through the Yidu Cloud electronic medical record retrieval system, and there was some selection bias and information bias. Secondly, some covariates were not included in the regression analysis model in this study, such as some known confounding factors that may influence the occurrence of HUA (e.g. intake of sugary soft drinks, exercise, tea consumption, high purine diet, etc.) that could not be collected through the electronic medical record system and may have some impact on the study results.

### Implications

By collecting and analyzing the clinical data of 3196 patients with undiagnosed HUA at baseline from 2010 to 2021 in Dalian Municipal Central Hospital of China, and by constructing a Cox proportional risk model, adjusting for relevant influencing factors, and further stratifying the analysis, this study clarified that smoking was an independent risk factor for the occurrence of HUA, and was independent of gender, whether a woman was menopausal, BMI and alcohol consumption, with large and innovative sample size. This study provides evidence that smoking is an independent risk factor for the occurrence of HUA. It has important implications for the prevention of HUA, but further prospective studies involving rigorous large samples are needed to further confirm the association between smoking and HUA.

## CONCLUSIONS

Smoking is an independent risk factor for the occurrence of HUA and is independent of gender, whether a woman is menopausal, BMI, and alcohol consumption. The smoking index ≥601 was an independent risk factor for the occurrence of HUA.

## Supplementary Material



## Data Availability

The data supporting this research are available from the authors on reasonable request.
