# Supplementary material for: The association between smoking and the occurrence of hyperuricemia: A retrospective cohort study
Source: Tob Induc Dis. 2025 May 30;23:10.18332/tid/204253. doi: 10.18332/tid/204253 (PMC12124120; doi:10.18332/tid/204253)
Supplement: Supplementary file 1 [file TID-23-73-s1.pdf]

The association between smoking and the occurrence of hyperuricemia: A retrospective cohort study

Supplementary file Table S1. Comparison of the incidence of HUA among groups with different gender smoking status, Dalian Municipal Central Hospital, China, between January 1, 2010, and January 1, 2021 (*N*=3196)

	Smoking status	*n* of HUA	*n*	Incidence of HUA	*P*
Total	Non-smoking	168	1400	12.0%	
	Smoking	252	1200	21.0%	<0.001*
	Smoking cessation	65	596	10.9%	0.486
Men	Non-smoking	75	587	12.8%	
	Smoking	190	854	22.2%	<0.001*
	Smoking cessation	54	497	10.9%	0.333
Women	Non-smoking	93	813	11.4%	
	Smoking	62	346	17.9%	0.003*
	Smoking cessation	11	99	11.1%	0.436

The χ^2 test was used for the comparison of rates. *P<0.05 is significant.

Supplementary file Table S2. Comparison of the incidence of HUA among groups with different smoking index, Dalian Municipal Central Hospital, China, between January 1, 2010, and January 1, 2021 (*N*=3196)

	Smoking index	*n* of HUA	*n*	Incidence of HUA	*P*
0 group	0	233	1996	11.7%	
1 group	1-200	37	193	19.2%	0.002*
2 group	201-400	56	272	20.6%	<0.001*
3 group	401-600	44	209	21.1%	<0.001*
4 group	≥601	115	526	21.9%	<0.001*
Total		485	3196	15.2%	

The χ^2 test was used for the comparison of rates. *P<0.05 is significant.

© 2025 Li P. et al.
